# Monte Carlo Method and GA-MLR-Based QSAR Modeling of NS5A Inhibitors against the Hepatitis C Virus

**DOI:** 10.3390/molecules27092729

**Published:** 2022-04-23

**Authors:** Wissal Liman, Mehdi Oubahmane, Ismail Hdoufane, Imane Bjij, Didier Villemin, Rachid Daoud, Driss Cherqaoui, Achraf El Allali

**Affiliations:** 1 African Genome Center, Mohammed VI Polytechnic University, Ben Guerir 43150, Morocco; wissal.liman@um6p.ma (W.L.); rachid.daoud@um6p.ma (R.D.); 2 Department of Chemistry, Faculty of Sciences Semlalia, BP 2390, Marrakech 40000, Morocco; mehdi.oubahmane@ced.uca.ma (M.O.); i.hdoufane@uca.ac.ma (I.H.); cherqaoui@uca.ac.ma (D.C.); 3 Institut Supérieur des Professions Infirmières et Techniques de Santé (ISPITS), Dakhla 73000, Morocco; imane.bjij@gmail.com; 4 Ecole Nationale Supérieure d’Ingénieurs (ENSICAEN) Laboratoire de Chimie Moléculaire et Thioorganique, UMR 6507 CNRS, INC3M, FR3038, Labex EMC3, Labex SynOrg ENSICAEN & Université de Caen, 14118 Caen, France; didier.villemin@ensicaen.fr

**Keywords:** chemoinformatics, drug discovery, molecular descriptors, QSAR, HCV, NS5A

## Abstract

Hepatitis C virus (HCV) is a serious disease that threatens human health. Despite consistent efforts to inhibit the virus, it has infected more than 58 million people, with 300,000 deaths per year. The HCV nonstructural protein NS5A plays a critical role in the viral life cycle, as it is a major contributor to the viral replication and assembly processes. Therefore, its importance is evident in all currently approved HCV combination treatments. The present study identifies new potential compounds for possible medical use against HCV using the quantitative structure–activity relationship (QSAR). In this context, a set of 36 NS5A inhibitors was used to build QSAR models using genetic algorithm multiple linear regression (GA-MLR) and Monte Carlo optimization and were implemented in the software CORAL. The Monte Carlo method was used to build QSAR models using SMILES-based optimal descriptors. Four splits were performed and 24 QSAR models were developed and verified through internal and external validation. The model created for split 3 produced a higher value of the determination coefficients using the validation set (R^2^ = 0.991 and Q^2^ = 0.943). In addition, this model provides interesting information about the structural features responsible for the increase and decrease of inhibitory activity, which were used to develop eight novel NS5A inhibitors. The constructed GA-MLR model with satisfactory statistical parameters (R^2^ = 0.915 and Q^2^ = 0.941) confirmed the predicted inhibitory activity for these compounds. The Absorption, Distribution, Metabolism, Elimination, and Toxicity (ADMET) predictions showed that the newly designed compounds were nontoxic and exhibited acceptable pharmacological properties. These results could accelerate the process of discovering new drugs against HCV.

## 1. Introduction

Hepatitis C Virus (HCV) has significantly affected the lives of infected patients over the last century, as a small proportion of them shed the virus naturally. Most infected individuals develop a spectrum of liver diseases ranging from mild inflammation to extensive liver fibrosis, cirrhosis, chronic hepatitis C, and hepatocellular carcinoma [1,2]. According to the statistical report of the World Health Organization (WHO), an estimated 58 million people were infected with hepatitis C in 2019, and approximately 300,000 deaths were caused by HCV [3]. HCV belongs to the Flaviviridae family, the genus Hepacivirus. It is a single-stranded RNA virus encoded by 9600 nucleotide bases. The HCV genome consists of the open reading frames (ORF) between the 5’ and 3’ conserved untranslated regions encoding three structural proteins (C, E1 and E2) and seven non-structural proteins (NS1, NS2, NS3, NS4A, NS4B, NS5A and NS5B) [4]. HCV strains are classified into eight major genotypes, with 86 subtypes identified to date [5]. For the past two decades, the standard therapy for HCV infection has been based on peginterferon and the antiviral nucleoside analog ribavirin. To date, approximately half of patients achieved a lower sustained virologic response (SVR) and suffered from undesired harmful effects such as cardiac-related problems, leukopenia, and thrombocytopenia [6]. Recently, many direct-acting antiviral (DAA) drugs have been authorized for the treatment of HCV infection with higher SVR rates (>90%), shorter duration, and fewer adverse effects compared with older treatment therapies [7,8]. These innovative therapies have revolutionized HCV medicine and delivered significant insights into curing HCV patients. In 2016, an affordable combination treatment with the new drug Ravidasvir was shown to be safe and effective, with exceptionally elevated cure rates [9]. The fight against HCV infection is not fully covered due to the high costs associated with the therapies and the emergence of mutant strains resistant to DAA drugs. These treatments target three nonstructural proteins: NS3/4A protease, NS5B polymerase, and NS5A protein, which are involved in the replication and assembly processes of the virus [7].

The NS5A receptor is a 478-amino acid phosphoprotein containing three structural domains (I, II, and III) that terminate in four complementary functional zones (A, B, C, and D). The NS5A protein interacts with other important viral proteins (NS4B, NS5B, NS3) and host cell proteins (cyclophilin A, kinases and others) to regulate viral replication and assembly [10,11]. Due to its critical role in HCV replication, NS5A has emerged as a potential therapeutic target for treating chronic HCV infection. Recently, computational drug design has emerged as a powerful technique that plays a pivotal role in drug development. The quantitative structure–activity relationship (QSAR), which links the structural features of molecules to endpoints, is an important part of cheminformatics. The QSAR approach is widely used to predict biological activities and the development of new lead compounds. Thus, the biological activity of new structures based on the developed model can be easily determined using the QSAR method without the need for experimental synthesis and biological testing [12]. Due to its predictive power, the QSAR approach could also eliminate molecules with undesirable properties at an early stage. Therefore, it reduces the cost, time, and error rate in developing new drug molecules.

Continuing our recent work on the development of new potent inhibitors targeting the NS4B receptor of HCV [13], we report here several QSAR models targeting NS5A. The current marketed anti-NS5A drugs have common structural features including C2 axial symmetry and the presence of methyl carbamates on both extremities. However, the symmetrical nature of anti-HCV agents is not essential for the inhibition of HCV, as reported by Nakamura et al. [14]. The QSAR models were built based on the structural features of asymmetrical NS5A derivatives with their potent inhibitory activity. The first model, based on Monte Carlo optimization, was applied to develop SMILES-based QSAR models that provide insights into the design of novel anti-HCV agents. The second model aims to confirm the prediction of inhibitory activity of the designed molecules using the genetic algorithm multiple linear regression (GA-MLR) technique. ADMET analysis was used to investigate and evaluate the drug-likeness properties of the newly designed inhibitors.

## 2. Results and Discussion

### 2.1. SMILES-Based QSAR Model

**Figure 1:**
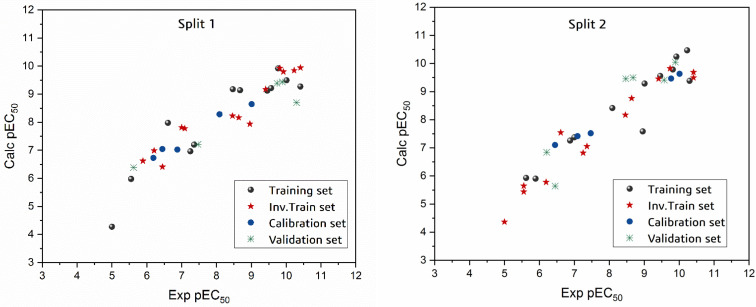

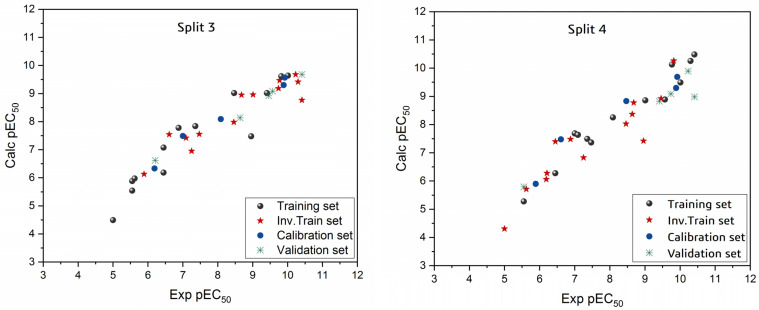
Experimental versus calculated pEC_50_ values for the models (i.e., Four Splits).

In total, 24 QSAR models were developed from four random splits using two objective functions: TF1 without the IIC and TF2 with different values of the IIC. For TF2, different numerical values of WIIC were used, including 0.1, 0.3, 0.5, 0.7, and 0.9. The calculated statistical parameters for the created SMILES-based QSAR models show that the WIIC = 0.5 strengthens the influence of IIC on the Monte Carlo optimization (Supplementary materials, Spreadsheet). The statistical parameters calculated with WIIC = 0.5 for all splits are shown in Table 1. The experimental pEC50 values compared to the calculated values for the four splits are shown in Figure 1. Table 1 clearly shows the statistical reliability of all models and that they meet the criteria established by Tropsha et al. [15] and Ojha et al. [16]. The established QSAR model of split 3 provides the best statistical parameters (*R*^2^ = 0.991, CCC = 0.911, and *Q*^2^ = 0.943). The model equation of split 3 is given below:

pEC_50_ = 0.532 (± 0.184) + 0.103 (± 0.003) × DCW (2,30) (1)

An additional validation model for the Monte Carlo method was performed using The AD. We determined the theoretical range in which the predictions of the constructed SMILES-based QSAR model are accurate. In the case of TF1, without considering the influence of IIC on activity (pEC50), the number of outliers for split 3 was four (i.e., compounds No. 29, 32, 34, and 35). In the case of TF2, the number of outliers for split 3 was three (i.e., compounds No. 8, 9, and 16).

### 2.2. GA-MLR QSAR Model

The GA-MLR method was performed on the training set and then evaluated against the test set based on the selected descriptors. In the GA-MLR model, the three selected descriptors from the entire set including **RBN** (i.e., No of rotatable bonds), **MATS1e** (i.e., Moran autocorrelation of lag 2 weighted by Sanderson electronegativity) and **G(N..O)** (i.e., Sum of geometrical distances between N..O), which contribute to the inhibition activity, were selected to build the QSAR model. The model created using the GA-MLR technique and its statistical parameters (Equation (2)) are shown below:

pEC50 = 7.078 − 0.105 × (**RBN**) + 43.362 × (**MATS1e**) + 0.013 × (**G(N..O)**) (2)

*N_tr_* = 26, *N_test_* = 10, $R_{tr}^{2}$ = 0.915, $RMSE_{tr}$ = 0.491, $Q_{loo}^{2}$ = 0.880, $R_{ext}^{2}$ = 0.941,

$MAE_{ext}$ = 0.416, $CCC_{ext}$ = 0.958, $Q_{F1}^{2}$= 0.914, $Q_{F2}^{2}$= 0.912, $Q_{F3}^{2}$= 0.920, *F* = 79.559, *s* = 0.534,

*Kxx* = 0.271, ∆K = 0.189, *RMSE_cv_* = 0.585, *RMSE_AV Yscr_* = 1.588, *R*^2^*_Yscr_* = 0.117, *Q*^2^*_Yscr_* = −0.269

where *N_tr_* is the total samples in training and *CCC* represents the concordance correlation coefficient [17]. $Q_{F1}^{2}$, $Q_{F2}^{2}$ and $Q_{F3}^{2}$ are external validation criteria [18].

**Figure 2:**
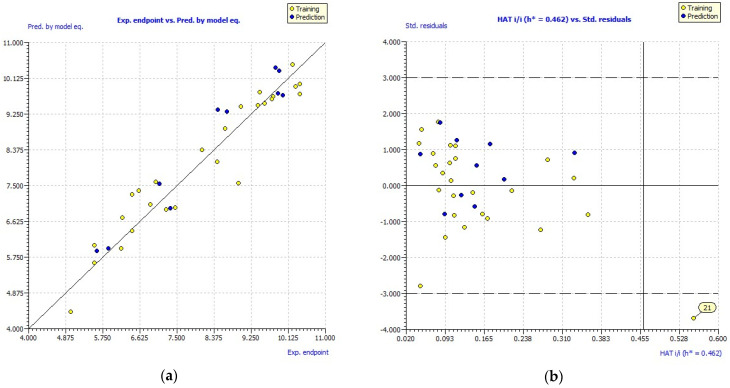
(**a**) Experimental vs. predicted pEC_50_ values computed by GA-MLR. (**b**) Williams plot.

The performance of the above parameters of the developed GA-MLR model meets the standard validation criteria according to the OECD guidelines. In addition, Figure 2a illustrates the experimental and the pEC50 endpoints predicted by the developed GA-MLR model, which shows a good correlation between the activity of concern and the three selected descriptors. To further validate the constructed model, the AD is used to evaluate the AD space of the leading model. The AD is performed with the leverage method as shown by the Williams plot in Figure 2b. The dashed lines show the cutoff value of ±3 s.d. and the warning line for the X outlier (h*) is 0.462. William plot show that all molecules are within the AD, with the exception of compound No. 21.

### 2.3. Mechanistic Interpretation

A mechanistic interpretation is a crucial part of OECD. Molecular features responsible for increasing and decreasing an endpoint can be extracted and interpreted from such models. The mechanistic interpretation of the CORAL model can be obtained from multiple runs of Monte Carlo optimization. In three independent Monte Carlo optimization runs, the molecular features extracted from the SMILES attributes with positive CWs are found to be promoters of an increase in pEC_50_ activity, and the SMILES attributes with negative CWs are found to be promoters of a decrease in pEC_50_ activity. In contrast, the SMILES attributes with both positive and negative CWs are undefined. The main promoters leading to an increase or decrease in pEC_50_ values with their CWs for three independent runs of the built QSAR model for split 3 are shown in Table 2.

Considering these data, the top-ranking fragments for increasing activity are: no. 1—combination of sp^3^ carbon with branching; no. 2—presence of sp3 oxygen surrounded by two sp^3^ carbons; no. 3—presence of oxygen; no. 4—combination of sp^3^ nitrogen with branching; no. 5—presence of sp^3^ carbon surrounded by sp^3^ oxygen and sp^3^ carbon; no. 6—combination of sp^3^ nitrogen and sp^3^ carbon in the aliphatic ring; no. 7—combination of sp3 nitrogen and sp3 carbon); no. 8—presence of sp^3^ nitrogen surrounded by two sp^3^ carbons; no. 9—presence of two sp3 carbon atoms; no. 10—presence of nitrogen; no. 11—presence of sp^3^ carbon surrounded by sp^3^ nitrogen and sp^3^ carbon; no. 12—maximum number of nitrogen is 8; and no. 13—maximum number of oxygen is 8. In contrast, the most ranking fragments for decreasing the activity are: no. 1—presence of one ring; no. 2—combination of oxygen, double bond, and branching; no. 3—presence of doubly bonded carbon; and no. 4—combination of sp^3^ oxygen and branching. Based on these considerations, the promoters of decrease were avoided. The promoters of propagation were exanimated in three different positions indicated by R1, L and R2 in the lead compound 25, which has the higher pEC50 value. The structures of all designed compounds with their pEC50 values are listed in Table S3 in the Supplemental Material.

**Figure 3:**
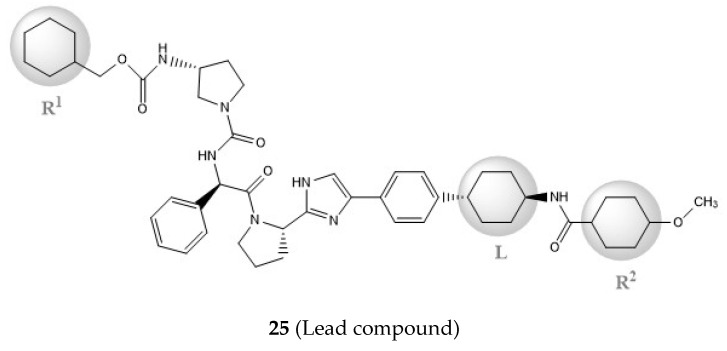

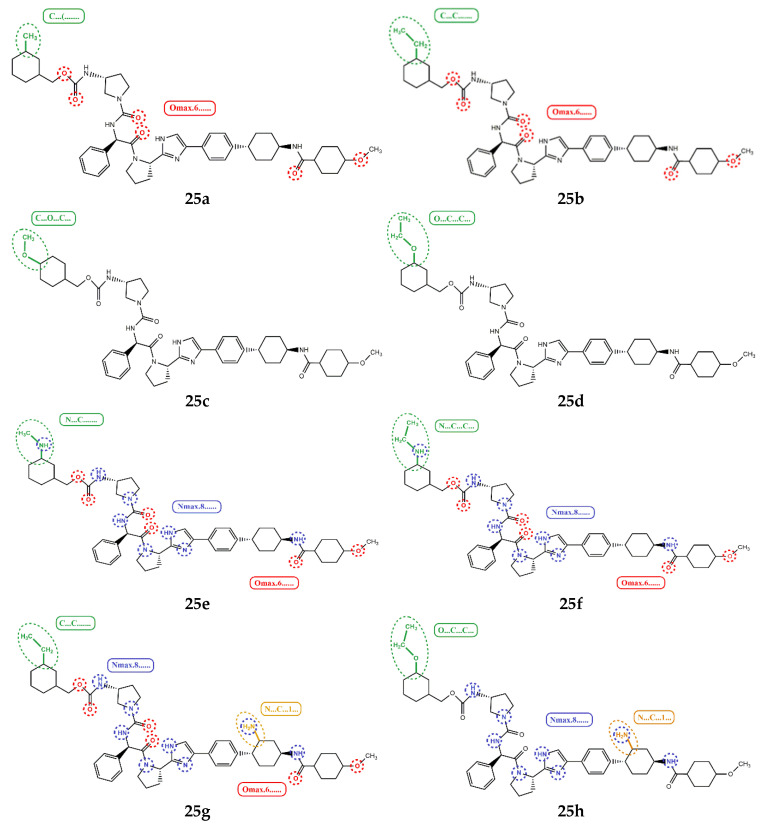
Chemical structures (**25a**–**25h**) of the newly designed compounds with favorable structural features.

Consequently, eight novel HCV NS5A inhibitors were selected based on these promoters, which showed high activity among the designed NS5A inhibitors (Figure 3). All pEC50 values of the selected inhibitors predicted by SMILES-based QSAR and GA-MLR QSAR models were higher than that of the lead compound 25 (Figure 3). These newly designed hits with their chemical structure, promoters increase, and predicted pEC50 values are shown in Figure 3 and Table 3.

### 2.4. ADMET Study

In silico ADMET analysis was performed using AdmetSAR and OSIRIS servers to evaluate the drug-likeness and pharmacokinetic characteristics of the newly designed compounds. The designed hit compounds do not present risks in terms of tumorigenic, irritant, mutagenic or reproductive effect profiles.

Water solubility is important for drug formulation and the determination of the persistence of organic compounds in the environment. The results in Table 4 show that all the newly developed compounds are soluble (water solubility is expressed in log (mol/L)). In addition, the blood–brain barrier (BBB) is the major interface between the central nervous system and the bloodstream. The BBB is an important property because it controls whether drugs can pass through the brain barrier and exert their effects. It is believed that a molecule with a logBB > −1 is widely distributed in the brain. Consequently, the BBB permeability results in Table 4 clearly show the non-penetrating BBB for the new suggested compounds. Moreover, intestinal absorption in humans (HIA) is one of the most important ADME properties. A compound with an intestinal absorption value greater than 30% is considered to be highly absorbed. Consequently, all newly developed compounds can be expected to have good biological activity, drug-like features, and ADMET properties.

## 3. Materials and Methods

### 3.1. Data Preparation

For this study, a dataset of 36 asymmetric inhibitors of HCV NS5A was used [14,19]. The chemical structures of these derivatives were drawn and were pre-optimized using the molecular mechanics’ force field MMFF94 of the ChemDraw package. Then, their geometries were optimized using the Gaussian 09 software [20], particularly the AM1 method in the gas phase. We calculated vibrational spectra to confirm the optimized structures to be the energy minima. The activity value of each molecule (half-maximal effective concentration, EC_50_) was converted to its negative logarithmic scale pEC_50_ = −log (EC_50_) and used as an independent variable to build QSAR models.

Two QSAR models were created using Monte Carlo optimization and the GA-MLR technique. For the Monte Carlo method, the simplified molecular input line entry system (SMILES) was used to symbolize the chemical structure and to develop QSAR models. They were generated with ACD/ChemSketch software (File Version C35E41, Build 125843, 14 Jan 2022, Toronto, ON, Canada) [21]. For the GA-MLR model, the molecular descriptor values (0D–3D) of the 36 compounds were computed using OCHEM [22]. To avoid multicollinear variables in the QSAR model, the total number of variables generated was reduced by excluding descriptors that possessed more than 95% constant values and descriptor pairs with a correlation coefficient greater than 0.9. A final set of 625 descriptors was selected from the initial pool of 3085 descriptors. The molecular structures and their corresponding pEC_50_ data are listed in Table 5 (the SMILES notation can be found in the Supplemental Materials in Table S1).

### 3.2. SMILES-Based QSAR Model Construction

The Monte Carlo optimization was used to create SMILES-based QSAR models using CORAL 2019 [23]. The SMILES attributes were used in this software to predict the endpoint using optimal descriptors (i.e., correlation weights (CWs)) and the balance-of-correlation method [24]. Four splits were created from the 36 compounds. Each split was randomly divided into 4 partitions: training (35%), invisible training (35%), calibration (15%), and validation (15%). Each set has a different task in constructing the QSAR model. The training set creates the QSAR model by calculating the correlation weight. The invisible training (inv. Train) set is assigned to evaluate the fitness of the molecules that are not included in the training set. The calibration set is used to identify the onset of overfitting, while the validation set is used to test the models for the compounds that are not included in the remaining sets [25,26,27].

Equation (3) describes the optimal descriptor of correlation weights:

^SMILES^DCW (*T*, *N*_epoch_) = ∑ CW(S_K_) + ∑ CW(SS_K_) + ∑ CW(SSS_K_) + CW (HARD) + CW (C_max_) + CW (N_max_) + CW (O_max_) (3)

^SMILES^DCW (*T*, *N*_epoch_) combines SMILES-based attributes associated with a correlation weight (CW). A description of the optimal SMILES parameters is provided in Table 6.

The linear regression approach was used to develop QSAR models after all CWs were calculated as shown in Equation (4).

pEC_50_= C_0_ + C_1_ × ^SMILES^DCW (*T*, *N*_epoch_) (4)

C_0_ is the intercept, while C_1_ is the slope of the regression equation.

In the Monte Carlo method, we defined *T* as the threshold and *N_epoch_* as the number of epochs. The *T* coefficient is used as a criterion to divide the SMILES attributes into two classes: an active class in which SMILES attributes are involved in model construction and a rare class (noise) that does not contain SMILES attributes. The *T* coefficient is used as a criterion to divide the SMILES attributes into two categories: an active class where SMILES attributes contribute to model construction and a rare class (noise) that contains no SMILES attributes. Overtraining can result from these rare attributes producing a good correlation during training and a poor correlation during validation. The *N_epoch_* provides the best statistical quality during calibration [28].

To develop the QSAR models, two types of target functions (TF) are used. TF1 uses balance of correlation as described in Equation (5), while TF2 adds the Index of Ideality of Correlation (IIC) described in Equation (6) [29,30]. IIC (Equation (7)) was proposed as a criterion for evaluating of the predictive power of the developed QSAR models. Namely, it improves the accuracy of the model measured by the coefficient of determination (R^2^) and the mean absolute error (MAE). The value of the coefficient WIIC (The weight of IIC) can change the strength of the influence of IIC on Monte Carlo optimization. The preferred value of WIIC can be determined by two factors: molecular diversity and endpoint nature [31,32,33].

(5) $${\mathrm{TF}}_{1}=R_{\mathrm{training}}+R_{\mathrm{inv}.\mathrm{train}}-\left|R_{\mathrm{training}}-R_{\mathrm{inv}.\mathrm{train}}\right|\times \mathrm{Const}$$

(6) $${\mathrm{TF}}_{2}={\mathrm{TF}}_{1}+\mathrm{IIC}\times W_{\mathrm{IIC}}$$

(7) $$\mathrm{IIC}=R_{\mathrm{set}}\times \frac{\min({}^{-}\mathrm{MAEset},{}^{+}\mathrm{MAEset})}{\max({}^{-}\mathrm{MAEset},{}^{+}\mathrm{MAEset})}$$

Rtraining and Rinv.train are correlation coefficients between the experimental pEC_50_ and the calculated pEC_50_ for each respective set. The empirical Const is typically fixed. Moreover, R_set_ is the value of the correlation coefficient between the observed and predicted endpoint of a give set. MAE is the mean absolute error, calculated as follows:

(8) $$\mathrm{MAEset}=\frac{1}{N\text{\_}}\sum_{k=1}^{N\text{\_}}\left|\Delta_{k}\right|\;\;\;\;(\Delta_{k}\;<\;0,\;{}^{-}N\;\mathrm{is}\;\mathrm{the}\;\mathrm{No}.\;\mathrm{of}\;\Delta_{k}<\;0)$$

(9) $${}^{+}\mathrm{MAEset}=\frac{1}{N_{+}}\sum_{k=1}^{N_{+}}\left|\Delta_{k}\right|\;\;\;\;(\Delta_{k}\;\geq \;0,\;{}^{+}N\;\mathrm{is}\;\mathrm{the}\;\mathrm{No}.\;\mathrm{of}\;\Delta_{k}\;\geq \;0)$$

(10) $$\mathrm{where},\;\Delta_{k}={\mathrm{Observed}}_{k}-{\;\mathrm{Predicted}}_{k}={\;\mathrm{pEC}}_{50}k\left(\mathrm{obs}\right)-{\;\mathrm{pEC}}_{50}k\left(\mathrm{pred}\right)$$

Δ_k_ is the accuracy for the *k*th substance from a set.

A grid-search was used for the best values of *T* and *N*_epoch_ for the four splits (1 to 10 for *T* and 1 to 30 for *N*_epoch_). The number of optimization probes was set to 3.

### 3.3. GA-MLR QSAR Construction

The first step in QSAR analysis is to choose the most relevant descriptors from the entire pool of computed descriptors. For this purpose, the stepwise linear regression method was applied, and the value of the leave-one-out cross-validation coefficient was used as the fitness function. Thus, 3085 different molecular descriptors were calculated using the OCHEM server [22]. The calculated descriptors were first examined to remove the near-constant and constant variables to decrease the redundancy in the matrix of descriptors. The correlation between the calculated descriptors and inhibitory activity was examined to exclude the collinear descriptors. Finally, 625 molecular descriptors were filtered out from the original set of variables. Then, the stepwise-MLR method was used to select the most relevant descriptors. Finally, three molecular descriptors were selected from the whole set. Based on the selected molecular descriptors, the MLR method used the ordinary least squares (OLS) algorithm to establish a linear relationship between the pEC50 endpoints of NS5A inhibitors and their molecular descriptors. QSARINS software was used to create the GA-MLR model [34,35]. The data set was randomly split into training (26 molecules) and testing (10 molecules) sets with a percentage distribution of 70% and 30%, respectively. The default parameters were used to build the GA-MLR models, except for: *subsets* = 1 to 5, *maximum generation* = 10,000 and *mutation probability* = 0.05.

### 3.4. QSAR Models Validation

The validation process is essential in QSAR to test the model’s suitability to make reliable forecasts of the modeled activity for new compounds with an unknown reaction. This process is considered one of the crucial steps to check the robustness, predictability and reliability of any QSAR model. Four steps are usually used to validate the constructed model, including (a) internal validation or cross-validation using the training set, (b) Y-randomization, (c) independent validation using the test set, and (d) applicability domain (AD) evaluation [36].

#### 3.4.1. Validation of GA-MLR QSAR Model

In the GA-MLR, the validity of the generated QSAR model was confirmed based on: internal validation using leave-many-out (LMO) and leave-one-out (LOO) procedures, Y-randomization, independent validation, and finally by checking the model AD. Moreover, thorough fulfillment of the respective thresholds for the statistical metrics proposed in the literature was evaluated [37]: the determination coefficient $R_{tr}^{2}$ ≥ 0.6, the Cross-validated $Q_{loo}^{2}$ ≥ 0.5, the determination coefficient obtained for the test set $R_{ext}^{2}$ ≥ 0.6, the root-mean square error $RMSE_{tr}$ < $RMSE_{cv}$, the concordance correlation coefficient (CCC) ≥ 0.80, $Q_{Fn}^{2}$ ≥ 0.6, the Y-scramble correlation coefficient $R_{Yscr}^{2}$ < 0.2, the the Y-scramble cross-validation coefficient $Q_{Yscr}^{2}$ < 0.2, $Q_{Yscr}^{2}$ < $R_{Yscr}^{2}$, the root-mean-square of Y randomization $RMSE_{AV\;Yscr}$ and the mean absolute error (MAE) should be near to zero.

#### 3.4.2. Validation of CORAL QSAR Model

In Monte Carlo optimization, additional parameters were used to verify the quality of the predictions of the QSAR models. $C_{R_{p}^{2}}$ is the deviation of the mean determination coefficient of the randomized models ($R_{r}^{2}$) from the determination coefficient of the non-randomized models (R^2^). $C_{R_{p}^{2}}$ should be greater than 0.5 for an acceptable QSAR model. $R_{m}^{2}$ is a metric proposed by Roy et al. [38,39] to indicate the external predictability of QSAR models; the average $R_{m}^{2}$ ($AvgR_{m}^{2}$) should be greater than 0.5, and $\Delta R_{m}^{2}$ should be less than 0.2 ($\Delta R_{m}^{2}$ = $R_{m}^{2}$ (x,y) − $R_{m}^{2}$ (y,x). x is the experimental value while y is the predicted value of endpoint).

Any QSAR model that does not meet the above criteria is eliminated. The formulas for calculating these statistical parameters are listed in Supplementary Material Table S2.

### 3.5. Applicability Domain

The applicability domain (AD) was proposed by the Organization for Economic Cooperation and Development (OECD) guidelines. AD allows the evaluation of the uncertainty in the prediction of a given molecule based on its similarity to the compounds used to develop the model. Compounds outside the AD are considered as outliers

In CORAL QSAR models, the AD is determined by the calculated statistical defects d(A) of SMILES based on the distribution of available data among all sets (Equation (11)). The d(A) of the SMILES attribute is depicted as the difference between the probability of the attribute in the training set and that of the calibration set. Outliers are SMILES, whose SMILE error is higher than twice the average error over training set compounds.

(11) $$\left(A\right)=\frac{\left|P\left(A\right)-P^{\prime }\left(A\right)\right|}{N\left(A\right)-N^{\prime }\left(A\right)}$$

Here, P(A) and P′(A) are the probabilities of the attributes (A) in the training and calibration set, respectively; N(A) and N′(A) are the numbers of times attribute (A) appears in the training and calibration set respectively.

The statistical defect (D) for a particular molecule is the total of the statistical defects, d(A), of all the attributes accessible in the SMILES notation.

(12) $$D=\mathrm{defect}\left(\mathrm{SMILES}\right)=\sum_{k=1}^{\mathrm{NA}}d\left(A\right)$$

(13) $$A\;\mathrm{molecule}\;\mathrm{is}\;\mathrm{considered}\;\mathrm{outlier}\;\mathrm{when}\;\;D\;>\;2\;\times \;\bar{D}$$

$\bar{D}$ is the average of the calculated D of training, inv. Train and calibration sets [40].

In the GA-MLR model, the William plot of standardized residual versus leverage was used to visualize the model AD. Reliable model predictions have leverage values framed between the critical leverage with ±3 standard deviations and lower than the warning leverage value h* of 0.48. Outliers are compounds that fall outside the horizontal reference lines on the plot. In contrast, the influential chemicals are compounds that have h > h* [41].

### 3.6. ADMET Study

ADMET assessment is critical in the early phase of drug discovery. A high-quality therapeutic agent is expected to have excellent efficacy against the target receptor and excellent ADMET properties at a therapeutic dose. Therefore, it is necessary to evaluate the pharmacokinetic profile of the Hit compounds to prevent subsequent drug failure [42]. Drug-likeness properties explain how a compound is distributed inside an organism and thus influence its pharmacological efficacy [43]. The ADMET predictions of the designed compounds were evaluated using AdmetSAR and Osiris property explorer [44,45].

## 4. Conclusions

Hepatitis C virus is a worldwide health problem that causes several life-threatening chronic liver diseases. Currently, there is no effective vaccine against hepatitis C, and treatment is still quite difficult. Computational methods have repeatedly proven useful in addressing the unique challenges of antiviral drug discovery. In this study, two QSAR models were developed to determine the quantitative relationship between anti-NS5A HCV biological activity and the molecular structure of a series of NS5A inhibitors. Two models were constructed using the GA-MLR and Monte Carlo optimization techniques. The results of the two models were in accordance with OECD guidelines. The model based on SMILES was used to evaluate the effects of the presence or absence of different molecular fragments on the biological activity studied. These results provided insights into the design of the eight novel NS5A inhibitors (against the NS5A target). The GA-MLR model confirmed the obtained inhibitory activities of the eight compounds. The ADMET study demonstrated that the designed molecules have advantageous chemical properties that provide promising inhibitory activity against NS5A.

## Figures and Tables

**Table 1:** Statistical parameter of built QSAR models and their corresponding equations.

Split	Set	*N*	*R* ^2^	CCC	*IIC*	*Q* ^2^	$Q_{F1}^{2}$	$Q_{F2}^{2}$	$Q_{F3}^{2}$	*s*	*MAE*	*F*	$C_{R_{p}^{2}}$	$AvgR_{m}^{2}$	$\Delta R_{m}^{2}$	Equation
1	Training	13	0.872	0.931	0.800	0.812				0.671	0.543	75	0.820			pEC_50_ = 0.655 (±0.344) + 0.101 (±0.004) × DCW(1,30)
	Inv.Train	13	0.899	0.921	0.565	0.867				0.580	0.469	98	0.845			
	Calibration	5	0.964	0.906	0.982	0.893	0.909	0.853	0.942	0.456	0.367	81	0.798	0.615	0.156	
	Validation	5	0.891	0.853	0.824	0.710					0.685			0.592	0.194	
2	Training	13	0.889	0.941	0.314	0.856				0.561	0.388	81	0.843			pEC_50_ = −0.094 (±0.234) + 0.084 (±0.002) × DCW(1,30)
	Inv.Train	13	0.937	0.962	0.519	0.915				0.517	0.392	166	0.905			
	Calibration	5	0.989	0.963	0.994	0.975	0.927	0.927	0.950	0.438	0.342	290	0.858	0.695	0.094	
	Validation	5	0.860	0.904	0.691	0.566					0.592			0.767	0.132	
3	Training	13	0.873	0.932	0.801	0.837				0.634	0.498	76	0.848			pEC_50_ = 0.532 (±0.184) + 0.103 (±0.003) × DCW(2,30)
	Inv.Train	13	0.865	0.871	0.581	0.829				0.685	0.510	71	0.805			
	Calibration	5	0.975	0.960	0.987	0.909	0.937	0.935	0.949	0.428	0.315	120	0.851	0.755	0.072	
	Validation	5	0.990	0.911	0.719	0.942					0.532			0.728	0.076	
4	Training	13	0.941	0.970	0.831	0.923				0.390	0.301	178	0.894			pEC_50_ = 0.457 (±0.157) + 0.149 (±0.003) × DCW(1,30)
	Inv.Train	13	0.843	0.914	0.601	0.801				0.650	0.483	59	0.807			
	Calibration	5	0.924	0.945	0.961	0.637	0.908	0.906	0.897	0.568	0.412	37	0.657	0.752	0.101	
	Validation	5	0.943	0.892	0.317	0.399					0.647			0.777	0.081	

*N*—number of samples; *s*—standard error of estimation; *F*—Fischer ratio.

**Table 2:** Promoters of increase and decrease of pEC_50_ endpoint from split 3.

No.	Sak	CWs Probe 1	CWs Probe 2	CWs Probe 3	N_T_ ^a^	N_iT_ ^b^	N_C_ ^c^	Defect [SAk] ^d^
Promoter of endpoint increase								
1	C...(.......	0.137	0.188	0.464	13	13	5	0.000
2	C...O...C...	2.041	1.674	2.516	6	8	3	0.015
3	O...........	1.351	1.397	1.810	13	13	5	0.000
4	N...(.......	0.054	0.063	0.110	13	13	5	0.000
5	O...C...C...	0.093	0.324	0.338	6	11	4	0.034
6	N...C...1...	0.222	0.101	0.043	7	6	3	0.006
7	N...C.......	0.423	0.479	0.573	13	13	5	0.000
8	C...N...C...	0.648	0.821	0.356	9	9	3	0.008
9	C...C.......	0.325	0.382	0.273	13	13	5	0.000
10	N...........	0.330	0.111	0.147	13	13	5	0.000
11	N...C...C...	0.798	0.643	0.829	11	13	5	0.009
12	Nmax.8......	0.798	0.510	0.590	2	2	0	1.000
13	Omax.6......	0.169	0.527	0.871	2	5	2	0.061
Promoter of endpoint decrease								
1	1...........	−0.1812	−0.0120	−0.0134	13	13	5	0.000
2	=...O...(...	−0.0579	−0.1326	−0.2212	13	13	5	0.000
3	C...=.......	−0.2642	−0.0515	−0.1487	13	13	5	0.000
4	O...(.......	−1.1001	−0.8219	−1.2695	13	13	5	0.000

N_T_, N_iT_, and N_c_ are the numbers of SMILES (samples) that include a given attribute (SAk) in the training set ^a^, inv.Training set ^b^, and calibration set ^c^. ^d^ Defect [SAk] is the difference of probabilities of SAk in the training and calibration sets, divided by the sum of total numbers of the SAk in the training and calibration sets.

**Table 3:** The newly designed compounds and their predicted pEC_50_ using the Monte Carlo optimization and the GA-MLR models.

Designed Compound	Promoters of Endpoint Increase	pEC_50_ (CORAL)	pEC_50_ (GA-MLR)
**25**		9.68	10.01
**25a**	- Combination of sp^3^ carbon with branching - Maximum number of oxygen is 6	9.88	10.15
**25b**	- Combination of two sp^3^ carbons - Maximum number of oxygen is 6	11.78	10.13
**25c**	- Presence of sp^3^ oxygen surrounded by two sp^3^ carbons	12.18	11.18
**25d**	- Presence of sp^3^ carbon surrounded by sp^3^ oxygen and sp^3^ carbon	12.27	11.23
**25e**	- Combination of sp^3^ nitrogen and sp^3^ carbon in aliphatic ring - Maximum number of oxygen is 6 - Maximum number of nitrogen is 8	11.95	10.71
**25f**	- Presence of sp^3^ carbon surrounded by sp^3^ nitrogen and sp^3^ carbon - Maximum number of oxygen is 6 - Maximum number of nitrogen is 8	12.05	12.28
**25g**	- Combination of two sp^3^ carbons - Combination of sp3 nitrogen and sp^3^ carbon in aliphatic ring - Maximum number of oxygen is 6 - Maximum number of nitrogen is 8	11.69	10.04
**25h**	- Presence of sp3 carbon surrounded by sp3 oxygen and sp3 carbon - Combination of sp3 nitrogen and sp^3^ carbon in aliphatic ring - Maximum number of nitrogen is 8	12.19	11.44

**Table 4:** Pharmacokinetic and ADME properties of the designed molecules and the lead compound evaluated using AdmetSAR and Osiris property explorer.

Pharmacokinetic Properties	MW (g·mol^−1^)	Lipophilicity (logP)	Solubility log(mol/L)	TPSA (Å^2^)	HBA	HBD	BBB	HIA
**25**	835.50	6.71	−2.88	157.99	13	4	0.012	0.010
**25a**	849.52	6.67	−3.11	157.99	13	4	0.018	0.010
**25b**	863.53	6.95	−3.20	157.99	13	4	0.015	0.009
**25c**	865.51	5.37	−3.32	167.22	14	4	0.009	0.012
**25d**	879.53	5.68	−3.49	167.22	14	4	0.009	0.007
**25e**	864.53	4.73	−3.12	170.02	14	5	0.013	0.189
**25f**	878.54	4.85	−3.06	170.02	14	5	0.010	0.078
**25g**	878.54	5.39	−3.06	184.01	14	6	0.023	0.049
**25h**	894.54	4.26	−3.28	193.24	15	6	0.015	0.035

Molecular weight (MW), blood–brain barrier (BBB), total polar surface area (TPSA), hydrogen bond acceptor (HBA), hydrogen bond donor (HBD), human intestinal absorption (HIA).

**Table 5:** Chemical structures and the studied biological activity data.

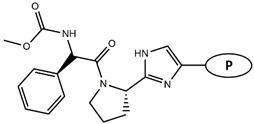				
**Compound**	**P**			**pEC_50_**
**1** ^t^	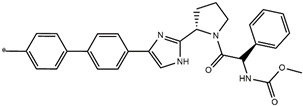			10.01
**2**	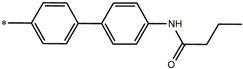			5.55
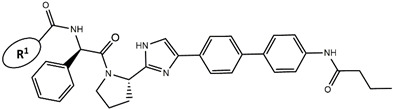				
**Compound**	**R^1^**			**pEC_50_**
**3** ^t^	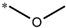			5.62
**4** ^t^	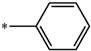			5.89
**5**	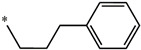			9.16
**6**	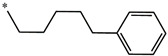			7.25
**7**	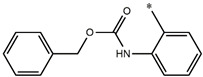			6.21
**8** ^t^	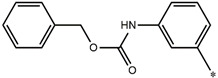			7.36
**9**	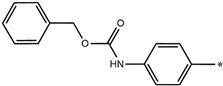			6.88
**10**	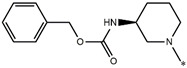			6.61
**11**	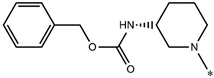			7.00
**12** ^t^	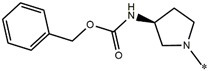			7.09
**13**	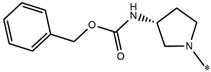			8.96
**14**	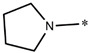			6.45
**15**	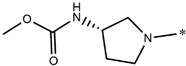			6.45
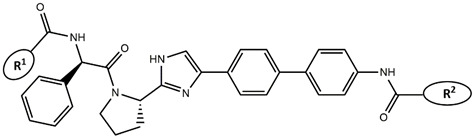				
**Compound**	**R^1^**		**R^2^**	**pEC_50_**
**16**	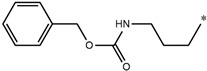		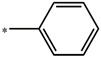	7.47
**17**	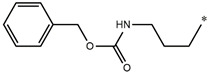		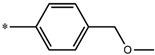	8.46
**18**	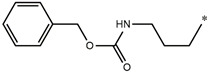		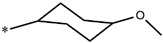	8.09
**19**	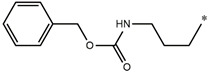		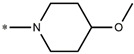	8.64
**20**	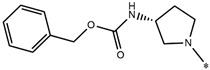		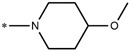	10.41
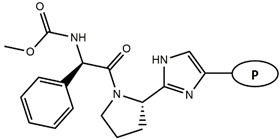				
**Compound**	**P**			**pEC_50_**
**21**	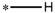			5
**22**	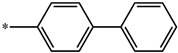			5.55
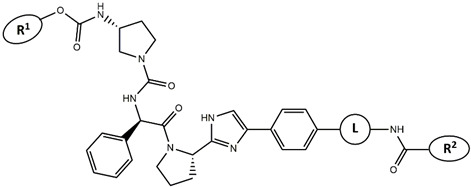				
**Compound**	**R^1^**	**L**	**R^2^**	**pEC_50_**
**23** ^t^	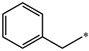	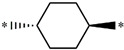	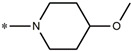	9.82
**24**	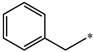	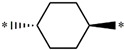	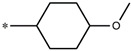	9.77
**25**	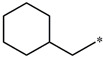	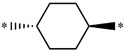	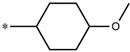	10.41
**26**	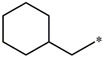	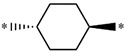	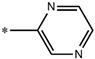	9.46
**27**	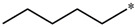	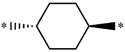	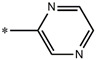	9.01
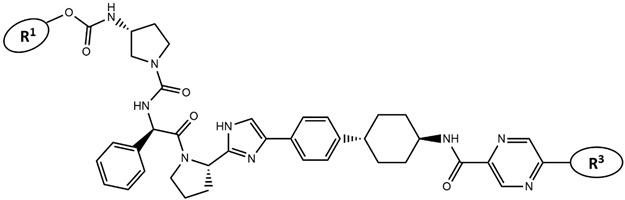				
**Compound**	**R^1^**	**R^3^**		**pEC_50_**
**28** ^t^	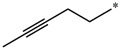	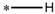		8.47
**29**	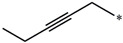	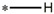		9.41
**30** ^t^	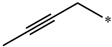	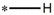		8.68
**31**	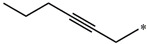	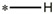		9.57
**32**	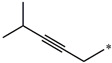	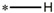		9.74
**33** ^t^	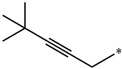	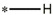		9.89
**34** ^t^	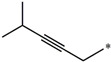	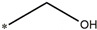		9.92
**35**	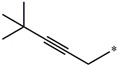	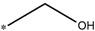		10.23
**36** (Daclatasvir)	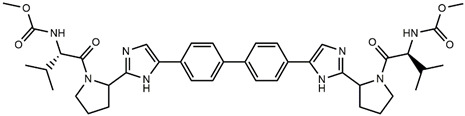			10.30

t: test set. *:Ramification position

**Table 6:** Description of the SMILES attributes.

SMILES Notation	Description
S_K_	One symbol or two symbols that cannot be examined separately
SS_K_	Combination of two SMILES atoms
SSS_K_	Combination of three SMILES atoms
HARD	Existence of some chemical element
C_max_	Number of rings
N_max_	Number of nitrogen atoms
O_max_	Number of oxygen atoms

## Data Availability

Not applicable.

## Supplementary Materials

The following supporting information can be downloaded at: https://www.mdpi.com/article/10.3390/molecules27092729/s1. Table S1: SMILES notation for the 36 compounds and their experimental activity data; Table S2: The mathematical equation of various statistical parameters; Table S3: Chemical structures of designed NS5A inhibitors.

## Sample Availability

Samples of the compounds are not available from the authors.
