# Supplementary material for: Monte Carlo Method and GA-MLR-Based QSAR Modeling of NS5A Inhibitors against the Hepatitis C Virus
Source: Molecules. 2022 Apr 23;27(9):2729. doi: 10.3390/molecules27092729 (PMC9099611; doi:10.3390/molecules27092729)
Supplement: Supplementary file 1 [file molecules-27-02729-s001.zip › molecules-1644506-supplementary.pdf]

Supporting Information

For

# Monte Carlo method and GA-MLR based QSAR modeling of NS5A inhibitors against the Hepatitis C Virus

Wissal Liman[1]; Mehdi Oubahmane[2]; Ismail Hdoufane[2]; Imane Bjij[3]; Didier Villemin[4]; Rachid Daoud[1] ; Driss Cherqaoui[2]; Achraf El Allali[1,*]

[1] African Genome Center, Mohammed VI Polytechnic University, Ben Guerir, Morocco.
[2] Department of Chemistry, Faculty of Sciences Semlalia, BP, 2390 Marrakech, Morocco.
[3] Institut Supérieur des Professions Infirmières et Techniques de Santé (ISPITS), 73000, Dakhla, Maroc.
[4] Ecole Nationale Supérieure d'Ingénieurs (ENSI CAEN) Laboratoire de Chimie Moléculaire et Thioorganique. UMR 6507 CNRS, INC3M, FR3038, Labex EMC3, Labex SynOrg ENSICAEN & Université de Caen, France.

* Corresponding author:

*E-mail addresse*: Achraf El Allali (Achraf.ELALLALI@um6p.ma)

## Table of contents

**Table S1**: SMILES notation for the 36 compounds and their experimental activity data .

**Table S2**: The mathematical equation of various statistical parameters.

**Table S3**: Chemical structures of designed NS5A inhibitors

**Table S1**: SMILES notation for the 36 compounds and their experimental activity data.

| ID | SMILES | $EC_{50}$ (nM) |
|---|---|---|
| 1 | COC(=O)N[C@@H](C(=O)N1CCC[C@H]1C1=NC(=CN1)C1=CC=C(C=C1)C1=CC=C(C=C1)C1=CNC(=N1)[C@H]1CCCN1C(=O)[C@@H](NC(=O)OC)C1=CC=CC=C1)C1=CC=CC=C1 | 0,098 |
| 2 | CCCC(=O)NC1=CC=C(C=C1)C1=CC=C(C=C1)C1=CNC(=N1)[C@@H]1CCCN1C(=O)[C@H](NC(=O)OC)C1=CC=CC=C1 | 2800 |
| 3 | CCCC(=O)NC1=CC=C(C=C1)C1=CC=C(C=C1)C1=CNC(=N1)[C@@H]1CCCN1C(=O)[C@H](NC(=O)C1=CC=CC=C1)C1=CC=CC=C1 | 2400 |
| 4 | CCCC(=O)NC1=CC=C(C=C1)C1=CC=C(C=C1)C1=CNC(=N1)[C@@H]1CCCN1C(=O)[C@H](NC(=O)CCCC1=CC=CC=C1)C1=CC=CC=C1 | 1300 |
| 5 | CCCC(=O)NC1=CC=C(C=C1)C1=CC=C(C=C1)C1=CNC(=N1)[C@@H]1CCCN1C(=O)[C@H](NC(=O)CCCCCCC1=CC=CC=C1)C1=CC=CC=C1 | 642 |
| 6 | CCCC(=O)NC1=CC=C(C=C1)C1=CC=C(C=C1)C1=CNC(=N1)[C@@H]1CCCN1C(=O)[C@H](NC(=O)CCCNC(=O)OCC1=CC=CC=C1)C1=CC=CC=C1 | 56 |
| 7 | CCCC(=O)NC1=CC=C(C=C1)C1=CC=C(C=C1)C1=CNC(=N1)[C@H]1CCCN1C(=O)C(NC(=O)C1=CC=CC=C1NC(=O)OCC1=CC=CC=C1)C1=CC=CC=C1 | 614 |
| 8 | CCCC(=O)NC1=CC=C(C=C1)C1=CC=C(C=C1)C1=CNC(=N1)[C@@H]1CCCN1C(=O)[C@H](NC(=O)C1=CC(NC(=O)OCC2=CC=CC=C2)=CC=C1)C1=CC=CC=C1 | 44 |
| 9 | CCCC(=O)NC1=CC=C(C=C1)C1=CC=C(C=C1)C1=CNC(=N1)[C@@H]1CCCN1C(=O)[C@H](NC(=O)C1=CC=C(NC(=O)OCC2=CC=CC=C2)C=C1)C1=CC=CC=C1 | 131 |
| 10 | CCCC(=O)NC1=CC=C(C=C1)C1=CC=C(C=C1)C1=CNC(=N1)[C@H]1CCCN1C(=O)[C@@H](NC(=O)N1CCC[C@H](C1)NC(=O)OCC1=CC=CC=C1)C1=CC=CC=C1 | 247 |
| 11 | CCCC(=O)NC1=CC=C(C=C1)C1=CC=C(C=C1)C1=CNC(=N1)[C@H]1CCCN1C(=O)[C@@H](NC(=O)N1CCC[C@@H](C1)NC(=O)OCC1=CC=CC=C1)C1=CC=CC=C1 | 100 |
| 12 | CCCC(=O)NC1=CC=C(C=C1)C1=CC=C(C=C1)C1=CNC(=N1)[C@@H]1CCCN1C(=O)[C@H](NC(=O)N1CC[C@@H](C1)NC(=O)OCC1=CC=CC=C1)C1=CC=CC=C1 | 82 |
| 13 | CCCC(=O)NC1=CC=C(C=C1)C1=CC=C(C=C1)C1=CNC(=N1)[C@@H]1CCCN1C(=O)[C@H](NC(=O)N1CC[C@H](C1)NC(=O)OCC1=CC=CC=C1)C1=CC=CC=C1 | 1.1 |
| 14 | CCCC(=O)NC1=CC=C(C=C1)C1=CC=C(C=C1)C1=CNC(=N1)[C@H]1CCCN1C(=O)[C@@H](NC(=O)N1CCCC1)C1=CC=CC=C1 | 353 |
| 15 | CCCC(=O)NC1=CC=C(C=C1)C1=CC=C(C=C1)C1=CNC(=N1)[C@H]1CCCN1C(=O)[C@@H](NC(=O)N1CC[C@@H](C1)NC(=O)OC)C1=CC=CC=C1 | 353 |
| 16 | O=C(CCCNC(=O)OCC1=CC=CC=C1)N[C@@H](C(=O)N1CCC[C@H]1C1=NC(=CN1)C1=CC=C(C=C1)C1=CC=C(NC(=O)C2=CC=CC=C2)C=C1)C1=CC=CC=C1 | 34 |
| 17 | COCC1=CC=C(C=C1)C(=O)NC1=CC=C(C=C1)C1=CC=C(C=C1)C1=CNC(=N1)[C@@H]1CCCN1C(=O)[C@H](NC(=O)CCCNC(=O)OCC1=CC=CC=C1)C1=CC=CC=C1 | 3.5 |
| 18 | COC1CCC(CC1)C(=O)NC1=CC=C(C=C1)C1=CC=C(C=C1)C1=CNC(=N1)[C@@H]1CCCN1C(=O)[C@H](NC(=O)CCCNC(=O)OCC1=CC=CC=C1)C1=CC=CC=C1 | 8.1 |
| 19 | COC1CCN(CC1)C(=O)NC1=CC=C(C=C1)C1=CC=C(C=C1)C1=CNC(=N1)[C@@H]1CCCN1C(=O)[C@H](NC(=O)CCCNC(=O)OCC1=CC=CC=C1)C1=CC=CC=C1 | 2.3 |
| 20 | COC1CCN(CC1)C(=O)NC1=CC=C(C=C1)C1=CC=C(C=C1)C1=CNC(=N1)[C@@H]1CCCN1C(=O)[C@H](NC(=O)N1CC[C@H](C1)NC(=O)OCC1=CC=CC=C1)C1=CC=CC=C1 | 0.039 |
| 21 | COC(=O)N[C@H](C(=O)N1CCC[C@@H]1C1=NC=CN1)C1=CC=CC=C1 | 10000 |

| | | |
|---|---|---|
| 22 | COC(=O)N[C@@H](C(=O)N1CCC[C@H]1C1=NC(=CN1)C1=CC=C(C=C1)C1=CC=CC=C1)C1=CC=CC=C1 | 2800 |
| 23 | COC1CCN(CC1)C(=O)N[C@H]1CC[C@@H](CC1)C1=CC=C(C=C1)C1=CNC(=N1)[C@H]1CCCN1C(=O)[C@@H](NC(=O)N1CC[C@@H](C1)NC(=O)OCC1=CC=CC=C1)C1=CC=CC=C1 | 0.15 |
| 24 | COC1CCC(CC1)C(=O)N[C@H]1CC[C@@H](CC1)C1=CC=C(C=C1)C1=CNC(=N1)[C@H]1CCCN1C(=O)[C@@H](NC(=O)N1CC[C@@H](C1)NC(=O)OCC1=CC=CC=C1)C1=CC=CC=C1 | 0.17 |
| 25 | COC1CCC(CC1)C(=O)N[C@H]1CC[C@@H](CC1)C1=CC=C(C=C1)C1=CNC(=N1)[C@H]1CCCN1C(=O)[C@@H](NC(=O)N1CC[C@@H](C1)NC(=O)OCC1CCCCC1)C1=CC=CC=C1 | 0.039 |
| 26 | O=C(N[C@H]1CCN(C1)C(=O)N[C@H](C(=O)N1CCC[C@@H]1C1=NC(=CN1)C1=CC=C(C=C1)[C@H]1CC[C@@H](CC1)NC(=O)C1=CN=CC=N1)C1=CC=CC=C1)OCC1CCCCC1 | 0.35 |
| 27 | CCCCCOC(=O)N[C@H]1CCN(C1)C(=O)N[C@H](C(=O)N1CCC[C@@H]1C1=NC(=CN1)C1=CC=C(C=C1)[C@H]1CC[C@@H](CC1)NC(=O)C1=CN=CC=N1)C1=CC=CC=C1 | 0.97 |
| 28 | CC#CCCOC(=O)N[C@H]1CCN(C1)C(=O)N[C@H](C(=O)N1CCC[C@@H]1C1=NC(=CN1)C1=CC=C(C=C1)[C@H]1CC[C@@H](CC1)NC(=O)C1=CN=CC=N1)C1=CC=CC=C1 | 3.4 |
| 29 | CCC#CCOC(=O)N[C@H]1CCN(C1)C(=O)N[C@H](C(=O)N1CCC[C@@H]1C1=NC(=CN1)C1=CC=C(C=C1)[C@H]1CC[C@@H](CC1)NC(=O)C1=CN=CC=N1)C1=CC=CC=C1 | 0.39 |
| 30 | CC#CCOC(=O)N[C@H]1CCN(C1)C(=O)N[C@H](C(=O)N1CCC[C@@H]1C1=NC(=CN1)C1=CC=C(C=C1)[C@H]1CC[C@@H](CC1)NC(=O)C1=CN=CC=N1)C1=CC=CC=C1 | 2.1 |
| 31 | CCCC#CCOC(=O)N[C@H]1CCN(C1)C(=O)N[C@H](C(=O)N1CCC[C@@H]1C1=NC(=CN1)C1=CC=C(C=C1)[C@H]1CC[C@@H](CC1)NC(=O)C1=CN=CC=N1)C1=CC=CC=C1 | 0.27 |
| 32 | CC(C)C#CCOC(=O)N[C@H]1CCN(C1)C(=O)N[C@H](C(=O)N1CCC[C@@H]1C1=NC(=CN1)C1=CC=C(C=C1)[C@H]1CC[C@@H](CC1)NC(=O)C1=CN=CC=N1)C1=CC=CC=C1 | 0.18 |
| 33 | CC(C)(C)C#CCOC(=O)N[C@H]1CCN(C1)C(=O)N[C@H](C(=O)N1CCC[C@@H]1C1=NC(=CN1)C1=CC=C(C=C1)[C@H]1CC[C@@H](CC1)NC(=O)C1=CN=CC=N1)C1=CC=CC=C1 | 0.13 |
| 34 | CC(C)C#CCOC(=O)N[C@H]1CCN(C1)C(=O)N[C@H](C(=O)N1CCC[C@@H]1C1=NC(=CN1)C1=CC=C(C=C1)[C@H]1CC[C@@H](CC1)NC(=O)C1=CN=C(CO)C=N1)C1=CC=CC=C1 | 0.12 |
| 35 | CC(C)(C)C#CCOC(=O)N[C@H]1CCN(C1)C(=O)N[C@H](C(=O)N1CCC[C@@H]1C1=NC(=CN1)C1=CC=C(C=C1)[C@H]1CC[C@@H](CC1)NC(=O)C1=CN=C(CO)C=N1)C1=CC=CC=C1 | 0.059 |
| 36 | COC(=O)N[C@@H](C(C)C)C(=O)N1CCC[C@@H]1C1=NC(=CN1)C1=CC=C(C=C1)C1=CC=C(C=C1)C1=CNC(=N1)[C@@H]1CCCN1C(=O)[C@H](NC(=O)OC)C(C)C | 0.05 |

**Table S2**: Mathematical equations of statistical parameters used.

| No | Equation |
|---|---|
| 1 | $R^2_{tr} = 1 - \frac{\Sigma\ (Y_{obs} - Y_{pred})^2}{\Sigma\ (Y_{obs} - \bar{Y})^2}$ |
| 2 | $Q^2_{loo} = 1 - \frac{\Sigma\ (Y_{obs} - Y_{pred})^2}{\Sigma\ (Y_{obs} - \bar{Y})^2}$ |
| 3 | $R^2_{ext} = \left[\frac{\sum_{i=1}^{n_{ext}}(Y_{obs} - \bar{Y}_{obs})\ (Y_{pred} - \bar{Y}_{pred})}{\sqrt{\sum_{i=1}^{n_{ext}}(Y_{obs} - \bar{Y}_{obs})^2\ (Y_{pred} - \bar{Y}_{pred})^2}}\right]^2$ |
| 4 | $Q^2_{F1} = 1 - \frac{\sum_{i=1}^{n_{ext}}(Y_{obs} - Y_{pred})^2}{\sum_{i=1}^{n_{ext}}(Y_{obs} - Y_{train})^2}$ |
| 5 | $Q^2_{F2} = 1 - \frac{\sum_{i=1}^{n_{ext}}(Y_{obs} - Y_{pred})^2}{\sum_{i=1}^{n_{ext}}(Y_{obs} - Y_{ext})^2}$ |
| 6 | $Q^2_{F3} = 1 - \frac{\sum_{i=1}^{n_{ext}} \frac{(y_i - \hat{y}_i)^2}{n_{test}}}{\sum_{j=1}^{n_{ext}} \frac{(y_j - \hat{y}_{TR})^2}{n_{TR}}} = 1 - \frac{RMSEP^2}{S^2_{TR}}$ |
| 7 | $IIC = \mathrm{R}_{set} \times \frac{\min(^{-}MAEset,\ ^{+}MAEset)}{\max(^{-}MAEset,\ ^{+}MAEset)}$ |
| 8 | $R^2_m = r^2(1 - \sqrt{\lvert r^2 - r_0^2 \rvert})$ |
| 9 | $CCI_{cal} = \Sigma\Delta R_{j\ calc};\ \Delta R_{j\ calc} < 0$ |
| 10 | $CCC = \frac{2\sum_{i=1}^{n_{ext}}(Y_{obs} - \bar{Y}_{obs})\ (Y_{pred} - \bar{Y}_{pred})}{\sum_{i=1}^{n_{ext}}(Y_{obs} - \bar{Y}_{obs})^2 + \sum_{i=1}^{n_{ext}}(Y_{pred} - \bar{Y}_{pred})^2 + n_{ext}(Y_{obs} - \bar{Y}_{pred})^2}$ |
| 11 | $C_{R^2_p} = R\sqrt{(R^2 - R^2_r)}$ |
| 12 | $MAE = \frac{1}{n} \times \Sigma\lvert Y_{obs} - Y_{pred}\rvert$ |
| 13 | $RMSE = \sqrt{\frac{1}{n} \times \Sigma\ (Y_{obs} - Y_{pred})^2}$ |

**Table S3**: Chemical structures of designed NS5A inhibitors

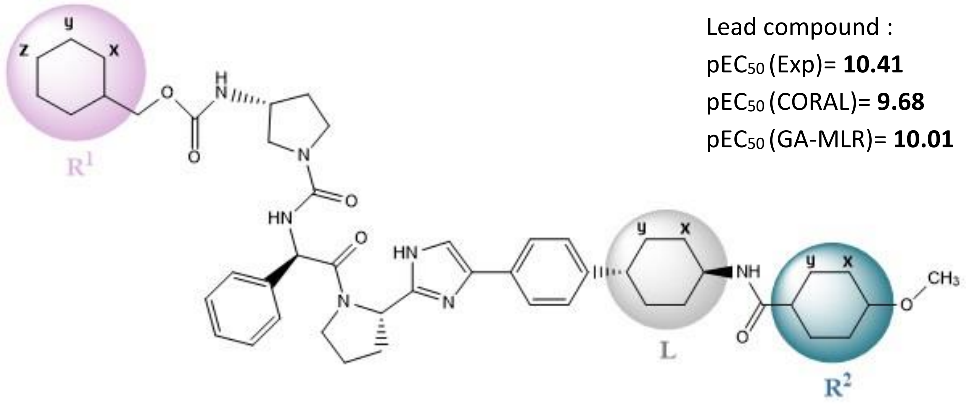


Lead compound :
$pEC_{50}$ (Exp)= **10.41**
$pEC_{50}$ (CORAL)= **9.68**
$pEC_{50}$ (GA-MLR)= **10.01**

| No. | | | Promoter of increase | Feature | $pEC_{50}$ (CORAL) | $pEC_{50}$ (GA-MLR) |
|---|---|---|---|---|---|---|
| 1 | $R^1$ | x | C...(....... | $-CH_3$ | 9.77 | 10.17 |
| 2 | | | C...C....... | $-C_2H_5$ | 9.75 | 10.16 |
| 3 | | | C...O...C... | $-OCH_3$ | 9.92 | 11.09 |
| 4 | | | O...C...C... | $-OC_2H_5$ | 10 .24 | 10.64 |
| 5 | | | N...C....... | $-NHCH_3$ | 9.23 | 10.63 |
| 6 | | | N...C...C... | $-NHC_2H_5$ | 10.03 | 11.11 |
| 7 | | y | C...(....... | $-CH_3$ | 9.88 | 10.50 |
| 8 | | | C...C....... | $-C_2H_5$ | 11.78 | 10.13 |
| 9 | | | C...O...C... | $-OCH_3$ | 9.95 | 11.20 |
| 10 | | | O...C...C... | $-OC_2H_5$ | 12.27 | 11.23 |
| 11 | | | N...C....... | $-NHCH_3$ | 11.95 | 10.71 |
| 12 | | | N...C...C... | $-NHC_2H_5$ | 12.05 | 10.70 |
| 13 | | z | C...(....... | $-CH_3$ | 9.84 | 10.17 |
| 14 | | | C...C....... | $-C_2H_5$ | 11.73 | 10.16 |
| 15 | | | C...O...C... | $-OCH_3$ | 12.18 | 11.18 |
| 16 | | | O...C...C... | $-OC_2H_5$ | 12.23 | 10.68 |
| 17 | | | N...C....... | $-NHCH_3$ | 11.91 | 10.67 |
| 18 | | | N...C...C... | $-NHC_2H_5$ | 12.01 | 11.16 |

| No. | | | Promoter of increase | Feature | $pEC_{50}$ (CORAL) | $pEC_{50}$ (GA-MLR) |
|---|---|---|---|---|---|---|
| 19 | L | x | C...(....... | —$CH_3$ | 9.95 | 10.14 |
| 20 | | | C...C....... | —$C_2H_5$ | 9.82 | 10.11 |
| 21 | | | C...O...C... | —$OCH_3$ | 9.29 | 10.75 |
| 22 | | | O...C...C... | —$OC_2H_5$ | 9.18 | 10.82 |
| 23 | | | N...C....... | —$NHCH_3$ | 10.03 | 10.53 |
| 24 | | | N...C...C... | —$NHC_2H_5$ | 9.35 | 10.52 |
| 25 | | y | C...(....... | —$CH_3$ | 9.45 | 10.14 |
| 26 | | | C...C....... | —$C_2H_5$ | 9.67 | 10.11 |
| 27 | | | C...O...C... | —$OCH_3$ | 9.98 | 10.70 |
| 28 | | | O...C...C... | —$OC_2H_5$ | 9.47 | 10.67 |
| 29 | | | N...C....... | —$NHCH_3$ | 9.56 | 10.49 |
| 30 | | | N...C...C... | —$NHC_2H_5$ | 9.78 | 10.47 |
| 31 | $R^2$ | x | C...(....... | —$CH_3$ | 9.95 | 10.17 |
| 32 | | | C...C....... | —$C_2H_5$ | 9.47 | 10.03 |
| 33 | | | C...O...C... | —$OCH_3$ | 10.03 | 11.31 |
| 34 | | | O...C...C... | —$OC_2H_5$ | 9.65 | 11.30 |
| 35 | | | N...C....... | —$NHCH_3$ | 9.74 | 10.72 |
| 36 | | | N...C...C... | —$NHC_2H_5$ | 9.96 | 10.71 |
| 37 | | y | C...(....... | —$CH_3$ | 9.82 | 10.17 |
| 38 | | | C...C....... | —$C_2H_5$ | 9.54 | 10.03 |
| 39 | | | C...O...C... | —$OCH_3$ | 9.98 | 11.19 |
| 40 | | | O...C...C... | —$OC_2H_5$ | 9.72 | 11.17 |
| 41 | | | N...C....... | —$NHCH_3$ | 9.81 | 10.66 |
| 42 | | | N...C...C... | —$NHC_2H_5$ | 10.05 | 10.65 |
